# Subcutaneous Injections

**Published:** 1871-05

**Authors:** 


Subcutaneous Injections. — Dr. Hanfield Jones recently presented to the Clinical Society of London a "Query as to the Safety of Subcutaneous Injections," in which three cases were recounted of more or less serious fainting after the injection of small doses of morphia or opium, and other published cases of serious symptoms following injections of morphia were cited. He thought it worthy of investigation whether intolerance of opium was manifested by any objective signs, and if such intolerance might exist at one time and not at another. He also asked whether valvular or muscular cardiac lesions increased the tendency of syncope, or whether, as seems to be thc case in chloroform syncope, some latent infirmity of the cardiac nervous centres is at fault.—*The Medical Gazette.*

